# Galanin is an epigenetically silenced tumor suppressor gene in gastric cancer cells

**DOI:** 10.1371/journal.pone.0193275

**Published:** 2018-02-20

**Authors:** Daseul Yoon, Kieun Bae, Min-Kyeong Lee, Jin Hee Kim, Kyong-Ah Yoon

**Affiliations:** 1 Department of Biochemistry, College of Veterinary Medicine, Konkuk University, Seoul, Korea; 2 Department of Cancer Biomedical Science, Graduate School of Cancer Science and Policy, National Cancer Center, Goyang, Korea; 3 Department of Biomedical Laboratory Science, College of Health Science, Cheongju University, Cheongju, Korea; Sapporo Ika Daigaku, JAPAN

## Abstract

Galanin is a 30 amino-acid active neuropeptide that acts via three G-protein coupled galanin receptors, GALR1, GALR2 and GALR3. Recently, GALR1 was also suggested as a tumor suppressor gene that was frequently silenced in head and neck squamous cell carcinoma; moreover, galanin and GALR1 were reported to inhibit human oral cancer cell proliferation. However, the exact role of galanin in gastric cancer is unclear. Here, we describe the epigenetic silencing of galanin in human gastric cancer. Five gastric cancer cell lines (SNU-1, SNU-601, SNU-638, KATOIII, and AGS) showed a significant reduction in galanin expression that was restored by the demethylating agent 5-aza-2’-deoxycytidine. We confirmed the hypermethylation of CpG islands in the galanin promoter region by methylation-specific and bisulfate sequencing polymerase chain reaction (PCR). Interestingly, hypermethylated *galanin* did not affect galanin receptor expression. Exogenous galanin expression in silenced cells induced apoptosis and decreased phosphorylated Akt expression. Taken together, these data suggest that galanin hypermethylation impairs its tumor suppressor function in gastric cancer carcinogenesis.

## Introduction

Galanin is a neuropeptide with a number of physiological actions that is distributed throughout the central and peripheral nervous systems. Human *galanin* is 6.6-kb long, with six exons that are translated into a 123 amino-acid prepropeptide; the galanin prepropeptide is then processed to active galanin and galanin message associated peptide [1]. Galanin signaling is mediated via three G-protein-coupled receptors: GALR1, GALR2, and GALR3 [2, 3]. Human galanin was isolated from the pituitary as a 30 amino-acid peptide with an additional non-amidated C-terminal residue [4]. Galanin has a variety of nervous system effects, including nociception, hormone regulation, and glucose metabolism [5, 6], and galanin and its receptors are also involved in cell proliferation and tumor progression. Galanin and GALR1 inhibited human oral cancer cell proliferation by down-regulating cyclin D1 and activating cyclin-dependent kinase inhibitors [7]. In addition, GALR1 is a candidate tumor suppressor that is frequently silenced in head and neck squamous cell carcinoma (HNSCC). Epigenetic inactivation of GALR1 was frequently found in HNSCC, and restoring GALR1 expression suppressed cellular proliferation with galanin [8]. GALR2 overexpression induced caspase-3 dependent apoptosis and inhibited cell proliferation in HNSCC cells that contained mutant p53 [9]. These previous results suggest that galanin receptors are feasible targets for novel cancer therapy. Inactivation and tumor-suppressive activity of galanin and its receptors were demonstrated mainly in HNSCC [10–12]. However, recent study reported galanin as a modulator for perineural invasion in head and neck cancer, suggesting its promotion of tumor progression, in contrast with previous studies [13]. However, the potential role of galanin in gastric cancer has not been sufficiently elucidated. Gastric cancer is the most common cancer, with a crude incidence of 79.2 per 100,000, and it is the third most common cause of cancer death among men in Korea [14]. It is important to identify target genes that are regulated by epigenetic regulation in gastric cancer. We examined the expression of galanin and its receptors in a number of gastric cancer cell lines and found frequent hypermethylation of CpG islands in the *galanin* promoter that correlated with its transcriptional silencing in gastric cancer. Furthermore, reintroducing galanin expression induced apoptosis, suggesting that galanin may have tumor suppressive effects in gastric cancer.

## Materials and methods

### Human gastric cancer cell lines and galanin expression

We purchased the human gastric cancer cell lines SNU-1, -5, -16, -216, -601, -638, -668, -719, AGS, and KATO III from the Korean Cell Line Bank [15, 16]. All cell lines were cultured in RPMI 1640 medium supplemented with 10% fetal bovine serum and antibiotics (Invitrogen, Carlsbad, CA, USA) at 37°C under 5% CO_2_.

We sub-cloned the coding region of the galanin prepropeptide was into pEGFP-N3 that contained the sequence for green fluorescent protein (GFP) (Clontech, Mountain View, CA, USA) or pcDNA3.1 vector (Invitrogen). Sequence was confirmed by direct sequencing and compared with reference mRNA sequence NM_015973. Vectors were transfected into cells using FuGENE (Roche, Mannheim, Germany), lipofectamine 2000 (Invitrogen), or electroporation (Gene Pulser Xcell™ Electroporation Systems, Bio-Rad, Hercules, CA, USA) according to the manufacturers’ protocols. Endogenous galanin expression was compared with glyceraldehyde-3-phosphate dehydrogenase (GAPDH) as a control with semi-quantitative RT-PCR.

### Methylation assay

To examine the methylation status of galanin, we performed methylation-specific PCR (MSP) using bisulfite-treated DNA from ten gastric cancer cell lines. DNA denaturation and bisulfite conversion were carried out using an EZ DNA methylation kit (Zymo Research, Foster City, CA, USA) according to the manufacturer’s instruction. The bisulfite-treated DNA was amplified with methylation-specific primers and unmethylation-specific primers as described in Table 1. We performed bisulfite sequencing PCR (BSP) to confirm the results of the methylation assay using the primers that we designed using MethPrimer (http://www.urogene.org/cgi-bin/methprimer/) [17]. For Sanger sequencing, PCR products were purified using a PCR purification kit (Qiagen, Redwood City, CA, USA) and then cloned into pCR2.1 TOPO vector (Invitrogen). At least ten clones were sequenced using T7 and M13 primers. We used universal methylated DNA (Chemicon International Inc., Temecula, CA, USA) as a positive control for MSP and BSP. Genomic DNA extracted from peripheral blood lymphocyte (PBLC) was used as a control.

**Table 1:** Primer sequences of galanin.

Galanin	Forward primer	Reverse primer
RT-PCR	5'-AAGGAAAAACGAGGCTGGAC-3'	5'-GGACCTGTCAAAGCTTCCTG-3'
Methylation- specific PCR	5'-GTTCGGATTTGTCGTTTAGATTCGTTATC-3'	5'-CGAAAACGCAAAA TCGACCCG-3'
Unmethylation- specific PCR	5'-TTGATGTTTGGATTTGTTGTTTAGATTTGTTATTG-3'	5'-CTCCCAAAAACACAAAATCAACCCA-3'
Bisulfite- sequencing PCR	5'-ATGATTTTTTAGTTGGGGTTG-3'	5'-AAAAAACCCCAAACCCTAT-3'

### Apoptosis and cell cycle analysis

One or two days after transfection, the cells were trypsinized and harvested for flow cytometric analysis. The cell cycle was analyzed following propidium iodide (PI) staining. We measured apoptosis using Annexin V conjugated to fluorescein isothiocyanate (FITC) (BD Pharmingen™, San Diego, CA, USA). Briefly, we fixed the harvested cells in 3% paraformaldehyde and ethanol and then stained them with annexin V reagent and PI. Flow cytometric analysis was carried out using the FACScaliber system (BD Biosciences, San Jose, CA, USA) or the NovoCyte flow cytometer system (ACEA Biosciences Inc., San Diego, CA, USA); the data were analyzed using Flowjo and NovoExpress software. All experiments were performed in triplicate and representative data were shown in figures.

### Western blot analysis

Harvested cells were lysed with RIPA buffer and quantified using a Qubit Protein Assay Kit (Invitrogen). Total cell lysates were subjected to SDS-PAGE and immunoblotting to detect exogenous Galanin with mouse monoclonal anti-GFP antibodies (Sigma-Aldrich, St. Louis, MO, USA). We also examined the levels of cleaved PARP and phosphorylated Akt (p-Akt serine 473; Cell Signaling Technology, Danvers, MA, USA). Polyclonal antibodies against phosphorylated Erk1/2(p-Erk1/2 threonine 202/ tyrosine 204), Erk1/2, cyclin D1 and horseradish peroxidase-linked secondary antibodies were also purchased from Cell Signaling Technology. Beta-actin was used as an internal protein loading control (Abcam, Cambridge, MA, USA). The results of western blotting analysis were demonstrated with representative examples from three independent experiments.

### Statistical analysis

Statistical significance was tested by unpaired t-test to compare measurements between groups using GraphPad Prism 5.0 (GraphPad, La Jolla, CA). P values for significant differences as calculated by unpaired two-tailed t-test are marked with * (p < 0.01), ** (p < 0.001), and *** (p < 0.0001) on figures, respectively.

## Results

### Decreased galanin expression in gastric cancer cell lines and its restoration by demethylating agent

**Fig 1:**
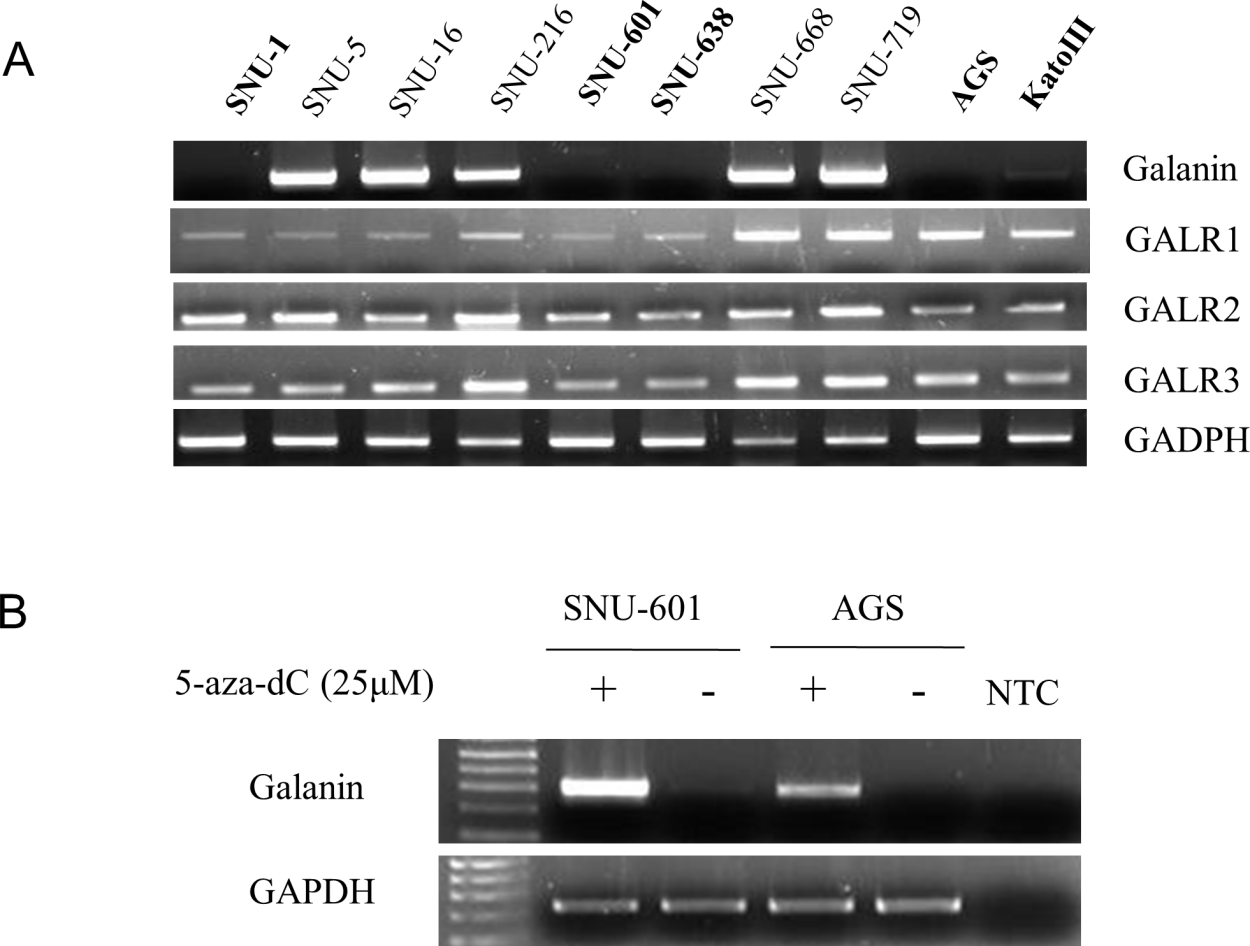
Decreased *galanin* expression and restoration by demethylating agent. (A) mRNA expression of *Galanin* and its receptors (GARL1-3) were examined by RT-PCR. (B) Recovery of *Galanin* mRNA expression was examined by RT-PCR following treatment with demethylating agent 5-aza-dC in SNU-601 and AGS cells.

To examine endogenous galanin expression, we performed RT-PCR in ten human gastric cancer cell lines (SNU-1, -5, -16, -216, -601, -638, -668, -719, AGS, and KATO III). Half of cell lines—SNU-1, SNU-601, SNU-638, AGS, and KATO III—showed down-regulated galanin expression. However, as shown in Fig 1A, galanin receptors expression (GALR1-3) was not altered in any of the cell lines.

To examine whether decreased galanin was associated with DNA methylation, demethylating agent was treated to galanin-silenced cell lines for recovery. Two cell lines showing complete down-regulation (SNU-601 and AGS) were treated with 25μM of the demethylating agent 5-aza-2’deoxycytidine (5-aza-dC or DAC). After 96 hours of treatment, galanin expression was rescued by 5-aza-dC in both cell lines as assessed by RT-PCR (Fig 1B). These data suggest epigenetic silencing of galanin in gastric cancer.

### Hypermethylation of galanin in gastric cancer

**Fig 2:**
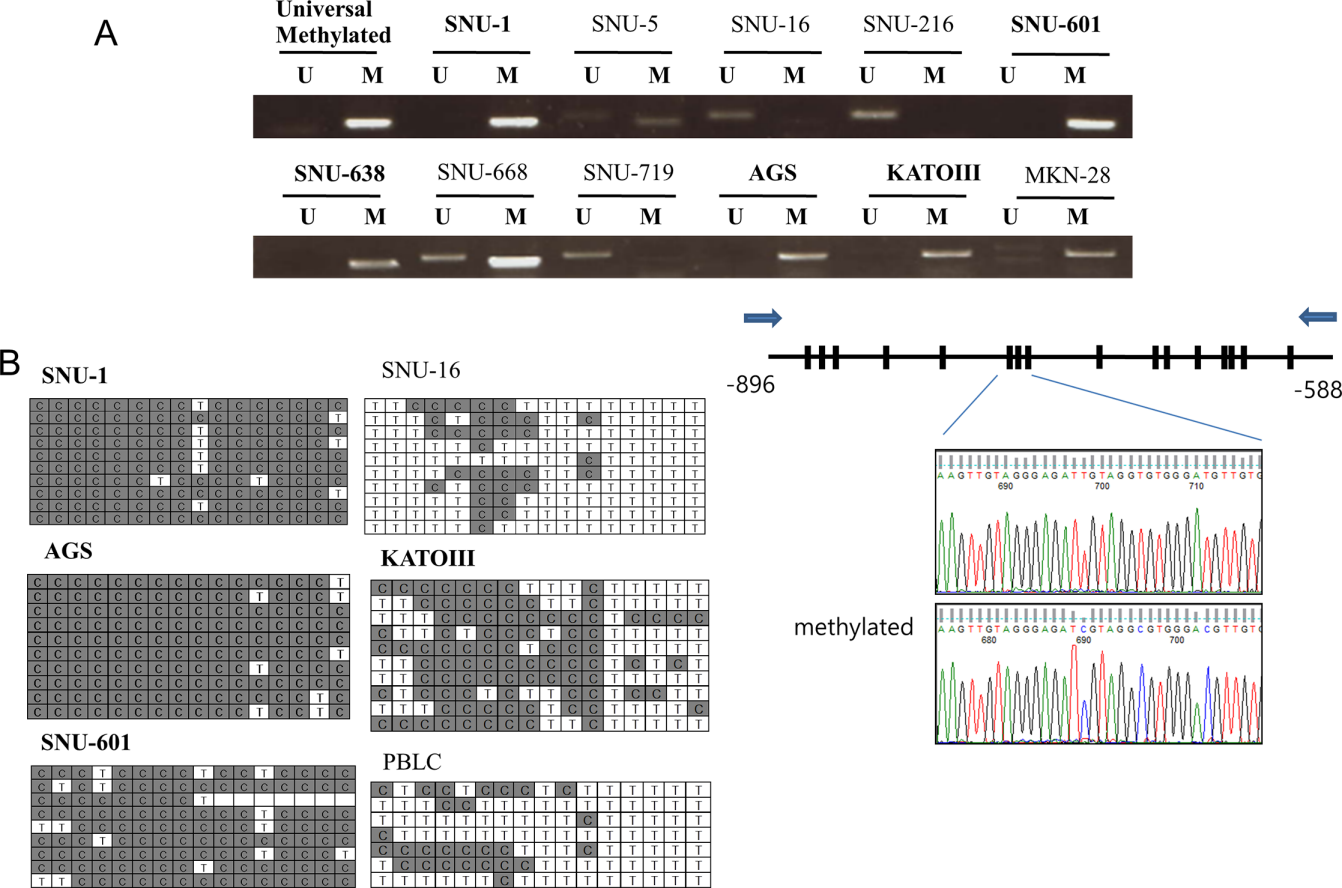
Methylation analysis of galanin in gastric cancer cell lines. (A) *Galanin* methylation status was analyzed by methylation-specific PCR (MSP) with methylation-specific primers (M) and ummethylation-specific primers (U). (B) Analysis of *galanin* hypermethylation by bisulfate-sequencing PCR (BSP). Schematic CpG island of galanin indicated 16 CpG dinucleotides (bars) in the genomic region that were examined using BSP primers (arrows). C and T represent methylated and unmethylated CpG islands, respectively. Representative chromatograms demonstrated methylated and unmethylated clones. PBLC is genomic DNA extracted from peripheral blood lymphocytes.

To determine the methylation status of galanin, MSP was carried out with bisulfite-modified DNA from gastric cancer cell lines. Predicted promoter region of galanin was amplified with methylation- and unmethylation-specific primers. Four of the lines (SNU-1, SNU-601, SNU-638, and AGS) were found to have methylated alleles but, three (SNU-16, SNU-216, and SNU-719) had no methylated alleles. The remaining three lines (SNU-5, SNU-668, and KATO III) possessed both types, but the alleles were predominantly methylated. Methylation pattern measured by bisulfite sequencing confirmed the results of MSP (Fig 2). Genomic region of upstream from the transcription start site of galanin was predicted to be a CpG island. As shown in Fig 2, the sequenced region harbors 16 CpG dinucleotide sites that are methylated in gastric cancers. The methylation patterns of 16 CpG sites matched the MSP results. SNU-1, AGS, and SNU-601cells showed highly methylated sites compared with PBLC.

### Overexpression of galanin in hypermethylated cell lines induces apoptosis

**Fig 3:**
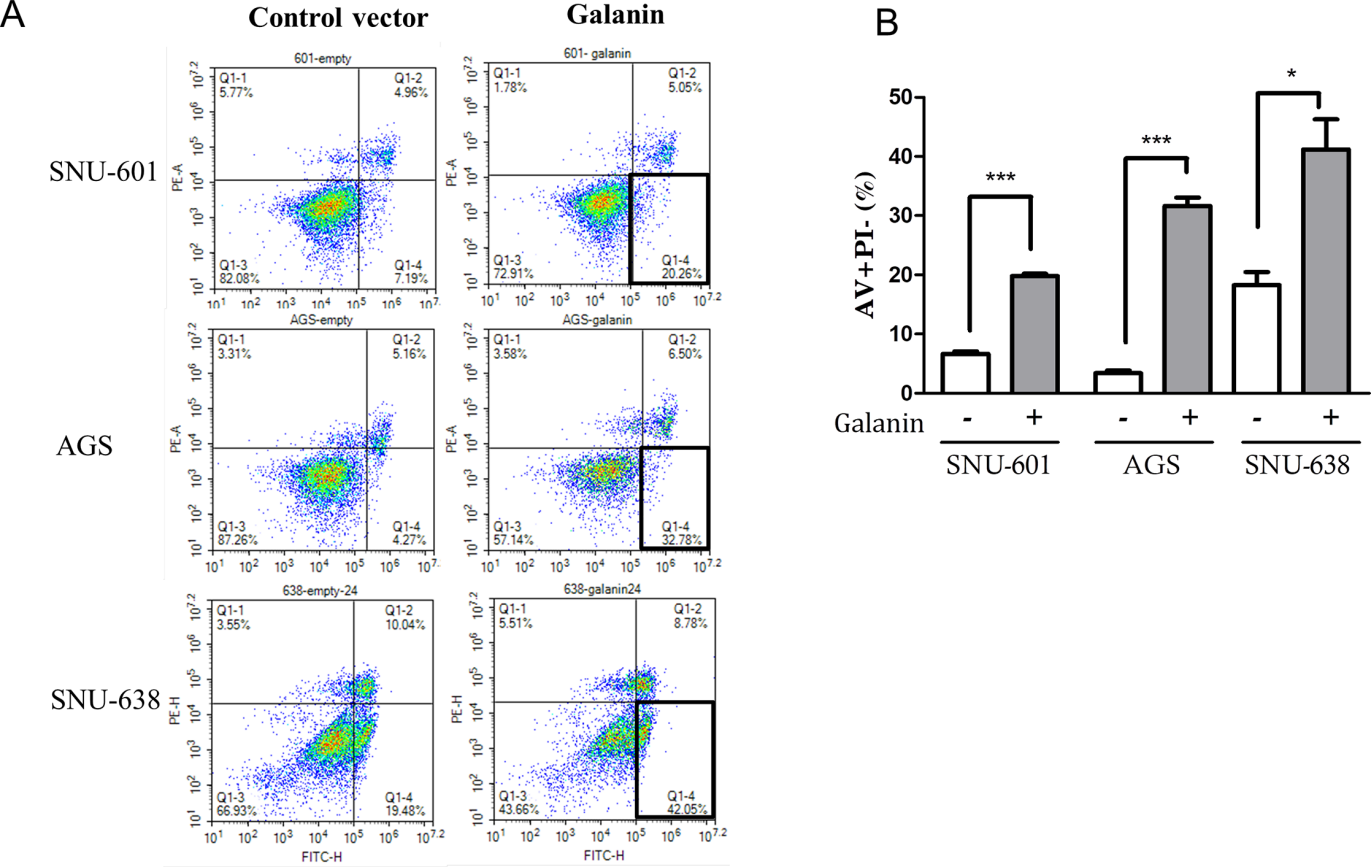
Induced apoptosis after galanin overexpression. (A) After 24 hours of transfection with each vector, apoptosis was analyzed by annexin-V and PI staining. Representative histograms from triplicate experiments show cells stained with annexin-V and propidium iodide. (B) Percentage of early apoptotic cells stained with only annexin-V (AV+PI-) are compared in three cell lines after galanin overexpression. Significant differences are marked with * (*p* < 0.01), *** (*p* < 0.0001), respectively, with mean ± standard error (SE).

**Fig 4:**
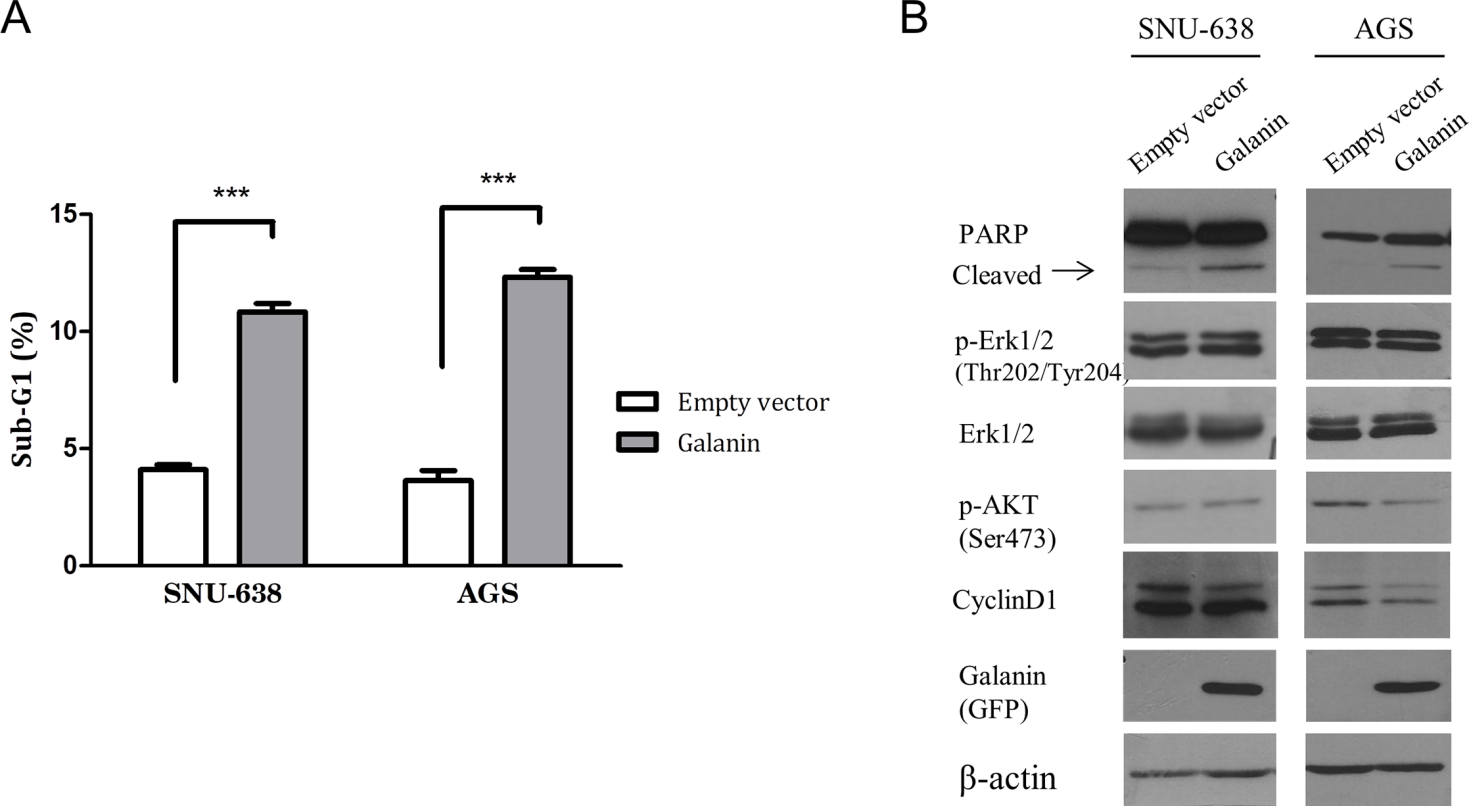
Apoptosis in galanin-overexpressing cell lines. (A) Sub-G1 population was compared between *galanin*- (pEGFP-Galanin) and empty vector (pEGFP)-transfected cells. Significant differences are marked with *** (*p* < 0.0001). (B) Protein level and phosphorylation were examined by western blot analysis. Enhanced PARP cleavage was shown in *galanin*-transfected cells (arrow).

We reintroduced exogenous galanin by transfecting pcDNA3.1_galanin vector into hypermethylated gastric cancer cell lines and analyzed apoptosis and cell cycle markers. Apoptosis was measured using annexin V staining, and annexin V positive and PI negative cells indicated early apoptosis. Early apoptosis increased dramatically in the cells that overexpressed galanin (Fig 3). Cell cycle analysis revealed that the sub-G1 population was significantly higher in the galanin-overexpressed cells compared with the controls 48 hours after transfection (Fig 4A). These results confirmed that overexpressed galanin could induce apoptosis in gastric cancer cells and supported the tumor suppressive effect of galanin. We also confirmed induced apoptosis by the increase of cleaved PARP in those cells by Western blotting analysis. Based on the previous studies that suggested the inhibitory effect of galanin on phosphorylated Akt, we examined the effects of galanin on Akt phosphorylation at serine 473 (p-Akt). Protein analysis revealed that phophorylated Akt and cyclin D1 had decreased slightly only in AGS cells after galanin transfection compared with the control cells (Fig 4B).

## Discussion

Epigenetic silencing plays an important role in carcinogenesis by inhibiting tumor suppressor gene expression [18, 19]. The extensive hypermethylation of CpG islands in gene promoter regions suppresses transcription and ultimately induces gene silencing or down-regulation. Hypermethylation of the CpG islands was one of the major factors underlying the inactivation of tumor suppressor genes such as *Rb*, *VHL*, *hMLH1*, and *BRCA1* in cancer.

Galanin is a neuropeptide found throughout the nervous system that exerts its effects via three G-protein coupled receptors: GALR1, GALR2, and GALR3. Previous studies on the role of galanin and its receptors in cancer have reported conflicting effects that vary according to cancer type. Galanin was associated with chromosome 11q13 amplification in breast cancer and squamous cell carcinoma and also reported to be a potential biomarker for colon cancer [20–22]. In contrast, anti-proliferative effects of galanin have been demonstrated in several types of tumors, including pheochromocytomas, pancreatic cancer, and gastric cancer [2, 23, 24]. As such, galanin and its receptors present attractive targets for novel cancer therapies. Recent studies have provided strong evidence supporting the tumor suppressive effects of galanin, GALR1, and GALR2 [7, 9, 25, 26], and they suggested that the inhibition of cell proliferation and apoptosis by galanin occurred via its receptors. Interestingly, *GALR1* was frequently hypermethylated in HNSCC, and it was shown to inhibit cell growth via ERK1/2 activation [8]. In this study, we hypothesized that promoter hypermethylation inactivates galanin prepropeptide expression in human gastric cancer cells. The presence of CpG islands in the region upstream of *galanin* made it feasible to examine whether *galanin* down-regulation was caused by epigenetic inactivation in gastric cancer. As far as we know, this study is the first report of epigenetic silencing of galanin in gastric cancer. We also examined galanin expression in seven lung cancer and five breast cancer cell lines, but we found the decreased expression in only one cell line (S1 and S2 Figs). We further reviewed galanin methylation data in cancer tissues from the Cancer Genome Atlas (TCGA) using MethHC database (http://methhc.mbc.nctu.edu.tw) [27]. The MethHC database revealed that gastric cancer as well as lung and breast cancers showed significantly higher promoter methylation of galanin compared with normal tissues (S3 Fig). Tofighi et al. [28] previously suggested that the PI3-kinase/AKT pathway mediated in galanin-exposed GALR2-overexpressing cells; however, decreased Akt phosphorylation was demonstrated in only AGS cells after galanin overexpression. The expression levels of galanin receptors including GALR2 were unaffected by galanin overexpression in AGS cells (S4 Fig). Given that exogenous galanin commonly induced cell death in silenced gastric cancer cell lines, further studies are needed to elucidate the precise mechanism of tumor suppressive function.

## Conclusions

In conclusion, we found that galanin down-regulation in gastric cancer cells was due to epigenetic inactivation. Furthermore, our data suggest that galanin hypermethylation abrogates its tumor suppressive properties in gastric cancer.

## Supporting information

S1 Fig Galanin expression and MSP in lung cancer cell lines. (PDF)

S2 Fig Galanin expression and MSP in breast cancer cell lines. (PDF)

S3 Fig Comparison of galanin methylation across tumors and normal tissues using MethHC database. (PDF)

S4 Fig Expression of galanin receptors in galanin overexpressed AGS cells. (PDF)
