# Supplementary figures and images for: Galanin is an epigenetically silenced tumor suppressor gene in gastric cancer cells
Source: PLoS One. 2018 Feb 20;13(2):e0193275. doi: 10.1371/journal.pone.0193275 (PMC5819827; doi:10.1371/journal.pone.0193275)

**S1 Fig. Galanin expression and MSP in lung cancer cell lines**

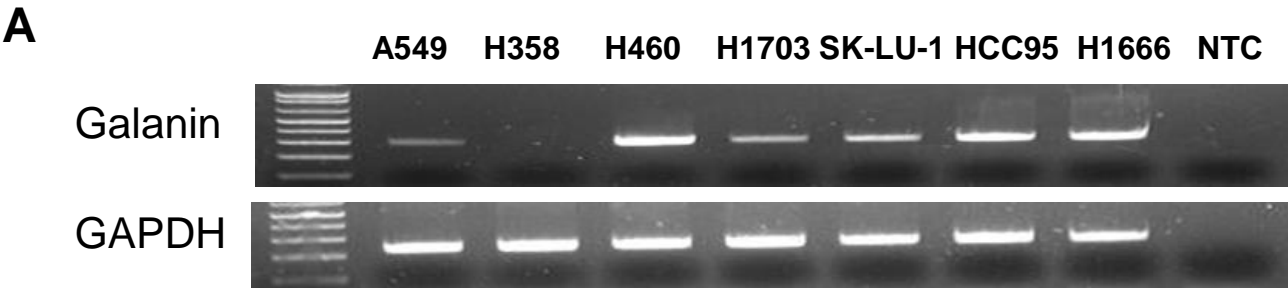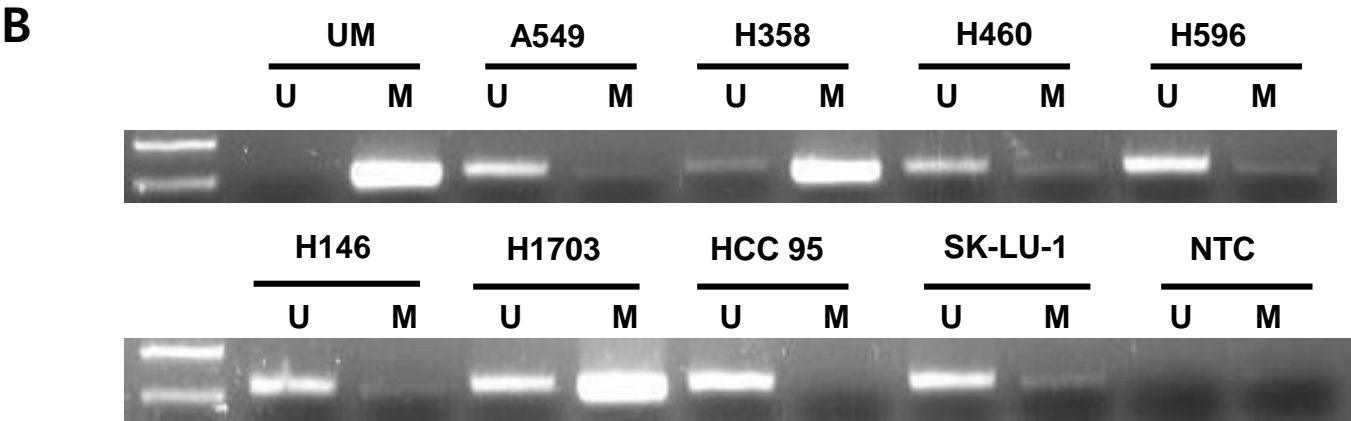

Supplement: S1 Fig — (PDF) [file pone.0193275.s001.pdf]

**S2 Fig. Galanin expression and MSP in breast cancer cell lines**

**A**

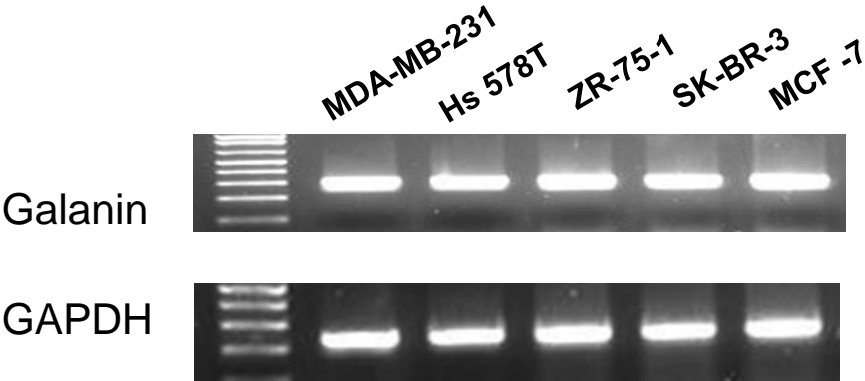

**B**

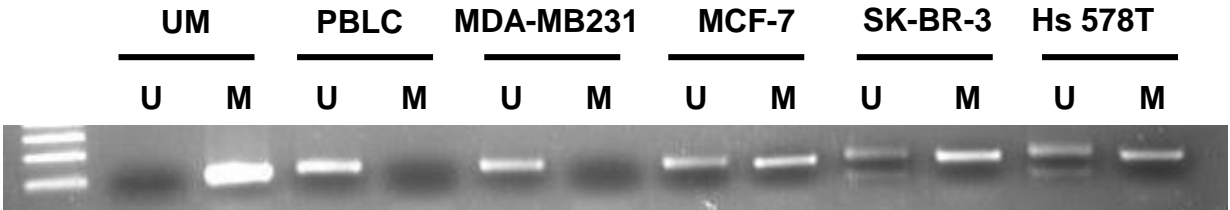

Supplement: S2 Fig — (PDF) [file pone.0193275.s002.pdf]

**S4 Fig. Expression of galanin receptors in galanin overexpressed AGS cells**

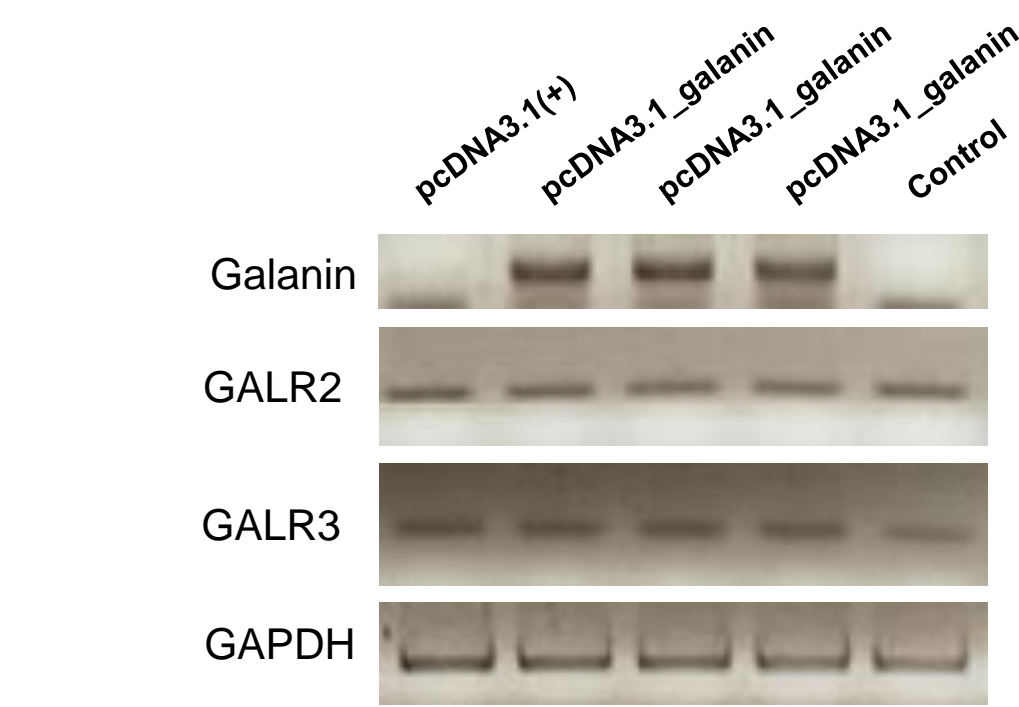

Supplement: S4 Fig — (PDF) [file pone.0193275.s004.pdf]
