# Supplementary material for: Galanin is an epigenetically silenced tumor suppressor gene in gastric cancer cells
Source: PLoS One. 2018 Feb 20;13(2):e0193275. doi: 10.1371/journal.pone.0193275 (PMC5819827; doi:10.1371/journal.pone.0193275)
Supplement: S3 Fig — (PDF) [file pone.0193275.s003.pdf]

S3 Fig. Comparison of galanin methylation across tumors and normal tissues using MethHC database

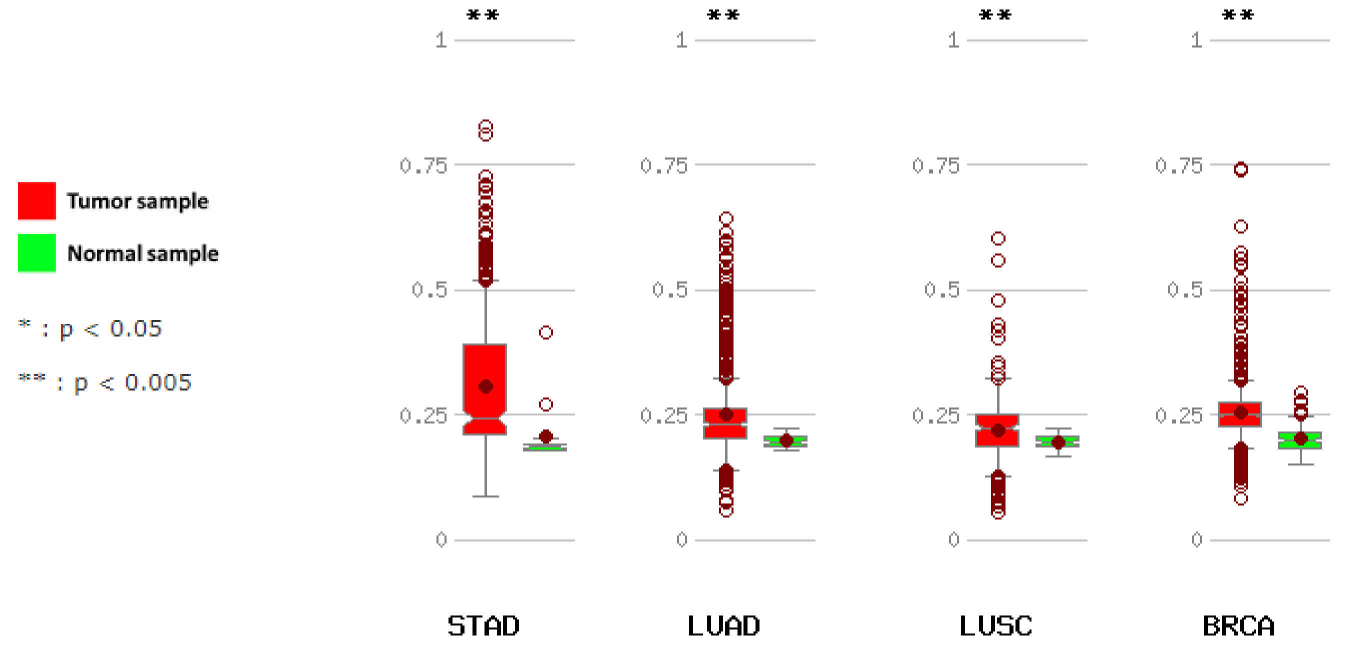

Cancer type		Gastric adenocarcinoma	Lung adenocarcinoma	Lung squamous cell carcinoma	Breast invasive carcinoma
P value		2.66E-08	1.73E-13	2.99E-11	2.29E-13
Cancer	No. of samples	326	452	361	748
	Beta value (Median)	0.242	0.230	0.224	0.249
Normal	No. of samples	15	48	59	129
	Beta value (Median)	0.185	0.195	0.195	0.198
